# Optimization of Pore Characteristics of Graphite-Based Anode for Li-Ion Batteries by Control of the Particle Size Distribution

**DOI:** 10.3390/ma16216896

**Published:** 2023-10-27

**Authors:** Yun-Jeong Choi, Young-Seak Lee, Ji-Hong Kim, Ji-Sun Im

**Affiliations:** 1 Hydrogen & C1 Gas Research Center, Korea Research Institute of Chemical Technology (KRICT), Daejeon 34114, Republic of Korea; yj6452@krict.re.kr; 2 Department of Chemical Engineering and Applied Chemistry, Chungnam National University, Daejeon 34134, Republic of Korea; youngslee@cnu.ac.kr; 3 Advanced Materials and Chemical Engineering, University of Science and Technology (UST), Daejeon 34113, Republic of Korea

**Keywords:** waste fine graphite, binder pitch, graphite block, porosity, lithium-ion battery anode, green manufacturing processes

## Abstract

We investigate the reassembly techniques for utilizing fine graphite particles, smaller than 5 µm, as high-efficiency, high-rate anode materials for lithium-ion batteries. Fine graphite particles of two sizes (0.4–1.2 µm and 5 µm) are utilized, and the mixing ratio of the two particles is varied to control the porosity of the assembled graphite. The packing characteristics of the assembled graphite change based on the mixing ratio of the two types of fine graphite particles, forming assembled graphite with varying porosities. The open porosity of the manufactured assembled graphite samples ranges from 0.94% to 3.55%, while the closed porosity ranges from 21.41% to 26.51%. All the assembled graphite shows improved electrochemical characteristics properties compared with anodes composed solely of fine graphite particles without granulation. The sample assembled by mixing 1.2 µm and 5 µm graphite at a 60:40 ratio exhibits the lowest total porosity (27.45%). Moreover, it exhibits a 92.3% initial Coulombic efficiency (a 4.7% improvement over fine graphite particles) and a capacity of 163.4 mAh/g at a 5C-rate (a 1.9-fold improvement over fine graphite particles).

## 1. Introduction

The demand for large-scale secondary batteries for electric vehicles and energy storage systems has rapidly increased. Consequently, there is increasing attention on high-rate and high-efficiency anode materials for use in lithium-ion batteries (LIBs) [1,2]. Accordingly, extensive studies are underway to improve various characteristics of materials, such as the rate performance and efficiency of natural graphite, which has been mostly utilized as an anode material for LIBs [3,4,5]. Graphite is the most frequently utilized anode material for LIBs owing to its high electrical conductivity, excellent reversibility for Li^+^ intercalation/deintercalation, and low operating potential [6,7]. In particular, natural graphite is considered a high-quality and cost-effective carbon material owing to its wide availability, better crystallinity than synthetic graphite, and low energy-intensive processes (thermal treatment is unnecessary) [8,9,10]. However, naturally mined graphite has large flake sizes, which results in limitations, such as Li^+^ diffusion inhibition and decreased electrode stability owing to accumulated Li^+^ (e.g., lithium dendrite formation) caused by particle orientation that occurs parallel to the current collector during the rolling process in electrode manufacturing [11,12,13]. Therefore, post-processing steps, such as grinding, classification, and spheronization, are essential to utilize natural graphite as an anode material. During post-processing, a substantial amount of fine graphite particles inevitably forms as by-products [14,15,16,17]. These fine graphite particles exhibit high purity and crystallinity, making them potential candidates for use in LIBs as anode materials. If these fine graphite particles can be utilized, they could resolve environmental and cost-related problems associated with waste disposal while improving the processing efficiency of graphite-based anode materials. However, to achieve this, the shape of the fine graphite particles must be modified to control particle orientation and reduce the specific surface area of the fine particles.

These problems can be addressed by binding fine graphite particles with binder materials to form assembled graphite (secondary particles) via granulation [15,18,19]. Granulation fixes the graphite crystalline to become poly-oriented with binder materials, facilitating multidirectional Li^+^ movement, and thus improving the rate performance [20,21]. However, existing studies have adopted complex procedures or equipment for manufacturing assembled graphite. In other words, additional processing costs to utilize low-grade graphite particle products have hindered their industrial application [16,22,23]. Therefore, studies should aim to establish methods to effectively use assembled graphite in existing anode manufacturing processes and apply fine graphite particle recycling technology to industrial applications.

In this study, assembled graphite with varying pore characteristics was produced by controlling only the particle size distribution of fine graphite particles. The adopted approach involves a relatively simple manufacturing process, primarily because only the ratio of the input materials in existing anode material production processes is required. Assembled graphite was prepared by mixing and shaping particle size-controlled fine graphite particles with a binder pitch. The pore characteristics of the manufactured assembled graphite were investigated by analyzing the bulk, apparent, and true densities to determine the quantity and shape of the pores. The optimal processing conditions (mixing ratio of fine graphite particles) to manufacture high-rate, high-efficiency anode materials were deduced from the correlation between pore and electrochemical characteristics.

## 2. Materials and Methods

### 2.1. Materials

Commercial natural graphite with a size similar to that of fine graphite particles, generated as a process by-product, was purchased (US Research Nanomaterials, Inc., USA). Two types of fine graphite particles were utilized to examine the effect on the particle size distribution of fine graphite particles: large and small particles. The average size of large particles (C > 99.5%, Ash: <0.5%; Moisture: <0.3%, CAS#7782-42-5) was 5 µm, whereas that of small particles (C > 99.9%, Ash: <0.5%; Moisture: <0.3%, CAS#7782-42-5) ranged from 0.4 to 1.2 µm. The two types of fine graphite particles were named NG5 and NG1.2, and both were flake-type graphite. A coal-tar pitch was used as the binder (Handan Jinghao Chemical Co., Ltd., Handan, China). The softening point and coking value of the binder were 107 °C and 34 wt%, respectively.

### 2.2. Experiments

**Figure 1:**
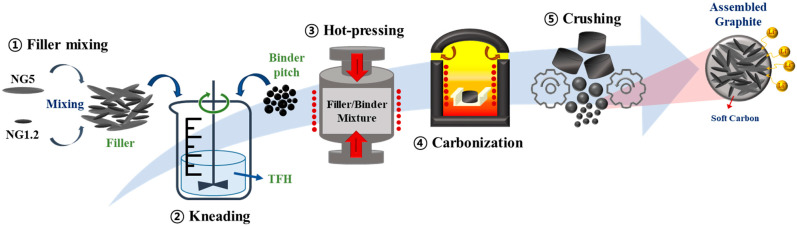
Schematic of the experimental method.

Assembled graphite was manufactured by bonding fine graphite particles with a binder. In this study, a block was manufactured with a constant state to improve the uniformity of the sample and the reproducibility of the experiment. The overall experimental schematic is shown in Figure 1. Blocks were manufactured through (1) filler mixing/kneading, (2) hot-pressing, and (3) carbonization. Fine graphite particles were used as the filler for the graphite block. The particle size distribution of the filler was set as a variable to control the particle packing in the assembled anode, which was controlled by mixing NG1.2 and NG5 by a weight ratio of 0:100, 10:90, 20:80, 30:70, 40:60, 50:50, 60:40, 80:20, and 100:0. Then, the mixed graphite filler was mixed with binder pitch at 70:30 vol%. The graphite and binder pitch mixing ratio was calculated from the true density to convert the weight format from the volumetric ratio. A total of 10 g graphite filler/binder mixture was kneaded with 30 mL tetrahydrofuran (THF) at 100 °C for 60 min to enhance the uniformity. The kneaded mixture was kept in a convection oven at 80 °C for 24 h to remove the residual THF. The mixture was put into a cylinder mold with a diameter of 10 mm and hot-pressed to 33.34 MPa (340 kgf/cm^2^) for 5 min at 120 °C (considering the softening point of the binder pitch) to prepare the green block. The green block was carbonized at 1300 °C for 60 min, and the carbonized block (graphite block) was named BSGXs (Xs; NG1.2 fraction). The manufactured graphite block was pulverized using a grinder (Mortar Grinder MG200, POWTEQ, Beijing Grinder Instrument Co., Ltd., Beijing, China) and then classified to 20 um or less using a sieve. Assembled graphite was obtained by granulation, as above, and the prepared anode sample was named SGXs (Xs; NG1.2 fraction).

### 2.3. Characterization of Assembled Anode

The tap density of the powder sample was measured by utilizing BT-301 (BETTERSIZE, Dandong, China). The tap density was calculated from the volume measured after 3000 taps at 300 taps/min, which was related to the packing density. The pore structure of the graphite block was predicted by utilizing three density values: bulk, apparent, and true densities. The bulk density was calculated from the diameter and height, measured using digital Vernier calipers. The underwater replacement method (JIS Z8807 [24]) was determined for the apparent density using a density meter (DME-220E, Shinko Denshi Co., Ltd., Tokyo, Japan). The true density was obtained by utilizing Micromeritics (AccuPyc II 1340 Pycnometer, Micromeritics, Norcross, GA, USA) in helium gas. In this study, the volume for bulk density was assumed to include open and closed pores, whereas that of the apparent density was assumed to only include closed pores. The true density was assumed to be the volume excluding the pores. The open pore, closed pore, and total porosity of the graphite block were calculated using Equations (1)–(3), as follows [25]:

(1) $$Total\;porosity\left(\%\right)=\left(1-\frac{Bulk\;density}{True\;density}\right)\times 100$$

(2) $$Open\;porosity(\%)=(1-\frac{Bulk\;density}{Apparent\;density})\times 100$$

(3) $$Closed\;porosity\left(\%\right)=Total\;porosity-Open\;porosity$$

The carbon yield of the graphite block was calculated as the remaining weight after carbonization at 1300 °C (weight after carbonization/before carbonization × 100, wt%). Images of the assembled graphite and electrode before and after the cycle were obtained via field emission scanning electron microscopy (FE-SEM) and energy-dispersive spectroscopy (EDS) analyses by using the Zeiss GemeniSEM 560 (Zeiss Group, Jena, Germany) at 10 kV. The specific surface area of the assembled graphite was measured by N_2_ adsorption at −196 °C, utilizing an ASAP 2420 (Micrometrics, Norcross, GA, USA), and calculated using the Brunauer−Emmett−Teller (BET) equation.

### 2.4. Electrochemical Characterization of Assembled Anode

Electrochemical measurements were performed using coin-type cells (CR2032-type). For the electrode, the slurry was mixed with a composition of 95 wt% active material, 2.5 wt% carboxymethylcelluloses, and 2.5 wt% styrene–butadiene rubber using a THINKY mixer (Thinky USA, Laguna Hills, CA, USA). Subsequently, the slurry was uniformly coated on a Cu current collector and dried at 80 °C for 8 h. The dried electrodes were punched into disk (∅ = 13.5 mm) forms. The loading level of the electrode was 5.0–5.2 mg/cm^2^, and the loading density was 1.38–1.39 g/cc. The porosity of the electrode was controlled at a range of 35–36% by roll-pressing. The coin cells were assembled in an Ar-filled glove box (H_2_O, O_2_ < 0.5 ppm). The electrolyte was 1.0 M LiPF_6_ with a mixture of ethylene carbonate and diethyl carbonate (1:1 vol%), and polyethylene was utilized as a separator. The galvanostatic charge–discharge of the electrode was measured at 25 °C in a voltage range of 0.01–1.5 V vs. Li/Li^+^ using a WonATech WBCS3000 (Seoul, Republic of Korea). To measure the rate performance, the coin cells were lithiated at a rate of 0.2 C and delithiated at various rates, ranging from 0.1 C to 5 C. Electrochemical impedance spectroscopy (EIS) was performed at an amplitude of 10 mV and in the frequency range of 100 kHz–0.01 Hz.

## 3. Results and Discussion

### 3.1. Effect of Graphite Filler Size Distribution on Block Porosity

**Figure 2:**
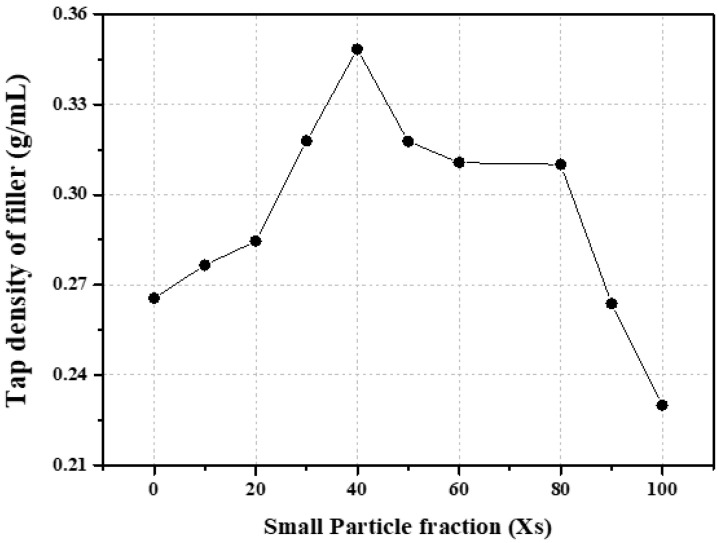
Tap density based on the fraction of small graphite particles.

Before the graphite block was manufactured, we measured the tap density of physically mixed fine graphite particles of two sizes (without a binder) to observe the packing characteristics based on their mixing ratios. The tap density measurements, corresponding to various fractions (Xs: 0, 10, 20, 30, 40, 50, 60, 80, 90, and 100%) of small graphite particles (NG1.2), are presented in Figure 2 and Table S1. Generally, when different sized particles are mixed, the smaller particles are packed between the larger particles, increasing the tap density [26]. To maximize the tap density, several factors, such as particle size and mixing ratio, must be considered simultaneously [26,27]. In this study, the maximum tap density was achieved when the fraction of small particles was 40%; the tap density exhibited a decreasing trend above and below this point. Based on these tap density measurements, five mixtures with maximum, minimum, and intermediate values in the increasing and decreasing tap density regions were selected to perform the granulation of fine graphite. The selected samples had Xs values of 0, 20, 40, 60, and 100, and their respective tap densities were 0.27, 0.28, 0.35, 0.31, and 0.23 g/mL, respectively.

**Figure 3:**
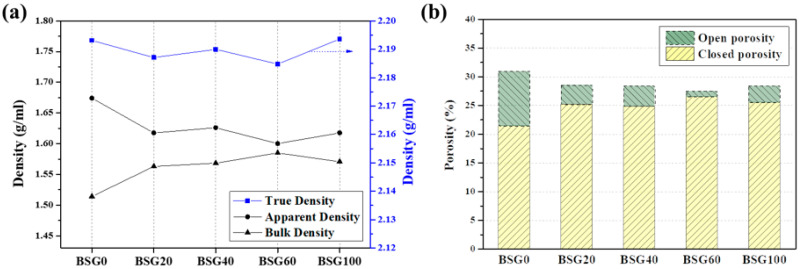
Density of the graphite block measured using various analysis methods and calculated porosity (**a**,**b**).

Graphite blocks (BSG-Xs) were prepared by mixing with the binder pitch, followed by kneading and carbonizing mixtures with Xs values of 0, 20, 40, 60, and 100. The packing characteristics of the manufactured graphite blocks were evaluated through three types of density (bulk, apparent, and true density), as presented in Table 1 and Figure 3a. All three densities showed a common trend reversal in BSG60. Such changes in density indicate a transformation in the pore structure. The porosity (open, closed, and total porosity) of the graphite block was calculated from the three types of density, as indicated in Table 1 and Figure 3b [25]. As the mixing ratio of smaller particles increased, total porosity decreased, reaching a minimum point in the BSG60 sample before increasing again. In other words, the BSG60 sample with a small particle mixing ratio of 60% represents the optimal point for porosity minimization, exhibiting the highest closed porosity (26.51%) and the lowest open porosity (0.94%).

**Figure 4:**
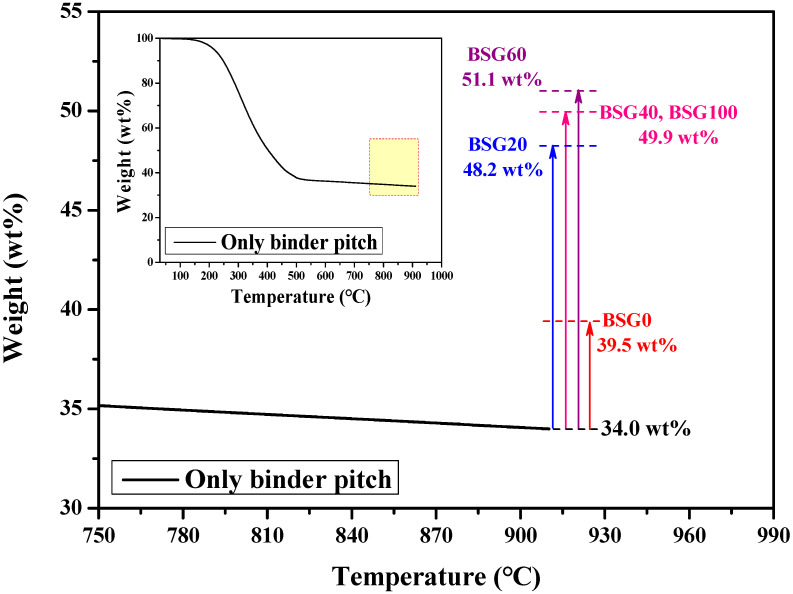
Difference in carbon yield after carbonization of the binder pitch only and after co-carbonization with a graphite block (inserted figure; thermogravimetric analysis curve only showing the carbonization of the binder pitch).

The total porosity and carbonization yield of the block were inversely related. Despite using the same type and ratio of binder pitch, the carbon yield between graphite blocks differed. As graphite has no mass or state changes during carbonization, only the carbonization behavior of the binder pitch influences the carbon yield of the graphite block. Assuming a 0% change in the mass of graphite during carbonization, the calculated carbon yields of the binder pitch in BSG0, BSG20, BSG40, BSG60, and BSG100 are 39.5, 48.2, 49.9, 51.1, and 49.9 wt%, respectively. This implies that the carbonization behavior of the binder pitch varied with the particle packing structure of the graphite filler. For an accurate comparison, we carried out TG analysis to investigate the carbon yield of the binder pitch carbonized without the graphite filler, as shown in Figure 4. Based on the results, the carbon yield of the binder pitch alone was 34 wt%, which is substantially lower than when co-carbonized with a graphite filler. Thus, the following conclusions can be drawn. First, when the binder pitch coexists with graphite filler, the interaction between graphite and pitch enhances the carbon yield of the binder pitch. Second, the particle packing structure of the graphite filler influences the carbonization behavior of the binder pitch, thereby altering the porosity of the graphite block.

**Figure 5:**
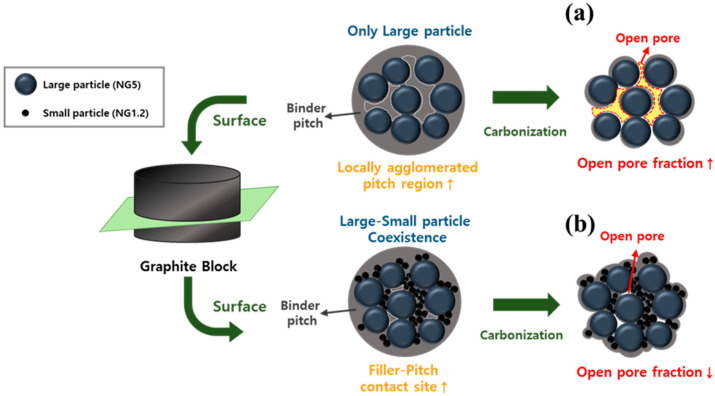
Correlation between the particle packing structure of the graphite filler and open porosity of graphite blocks (**a**,**b**).

The mechanism to explain these two facts is illustrated in Figure 5. Generally, the carbon yield of the pitch increases when it is co-carbonized with filler materials, such as cokes or graphite. This results from the interaction between the pitch and the filler material, which suppresses the volatilization of components with low boiling points in the pitch [28,29]. Therefore, the open porosity varied based on the contact area between the binder pitch and graphite filler, influenced by the volatilization of components with low boiling points. When only utilizing large particles as fillers to manufacture the block, the particle packing is loose, resulting in a relative increase in regions where the binder pitch locally aggregates. This increases the open pore fraction, as shown in Figure 5a, thereby decreasing the carbon yield. This explains the reason BSG0 exhibits the highest open porosity (30.96%) and the lowest yield (87.89 wt%). However, the contact sites between the filler and pitch increase when small particles are packed between large particles. In other words, the areas in which the pitch locally aggregates decreases. As shown in Figure 5b, this resulted in relatively low open porosity and high carbon yield.

Closed pores refer to internally isolated pores that are not connected to the exterior. Graphite blocks containing both small and large particles exhibited a high fraction of closed pores because the binder pitch was insufficient to fill the void spaces between particles. When the contact area of graphite filler increases, more binder pitch is required for bonding between particles. More binder pitch is required when containing both small and large particles because their contact area also increases. However, in this study, the same binder ratio prepared the entire graphite blocks. BSG 20, 40, and 60 are considered binder shortage conditions, rather than BSG0, in which a large portion of the pitch is used for the binding of particles [30]. Therefore, the effect of filling the void spaces between the particles can be weakened, and the void remains a closed pore [30].

For the high-density and high-strength graphite block, the formation of open and closed pores should be minimized by optimizing the amount of binder pitch in the highest tap-density filler particle mixture. However, from the perspective of electrode materials, the role of pores differs. As the ratio of open pores increases, the solid electrode interphase (SEI) layer forms broadly and unevenly, which should be minimized [31,32,33]. Conversely, closed pores formed inside graphite particles can act as a volume expansion buffer during repeated charge and discharge, improving long-term cycle stability [34,35]. The fine graphite assembly process presented in this study shows a phenomenon in which open pores transform into closed pores at the optimum point, suggesting that the SEI layer on the anode material will form stably while improving the the cycle stability.

### 3.2. Characteristics of Assembled Graphite According to the Porosity of the Graphite Block

**Figure 6:**
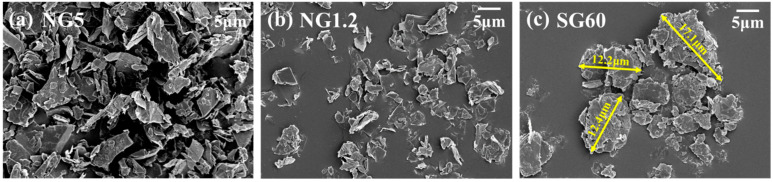
FE-SEM image of fine and assembled graphite via granulation; (**a**) NG5, (**b**) NG1.2, and (**c**) SG60.

The produced graphite blocks were ground and classified to sizes below 20 µm for application as anode materials in LIBs. The ground and classified assembled graphite particles were named SG-Xs. The images of the fine graphite particles utilized as a graphite filler and the assembled graphite (secondary particles by granulation) are shown in Figure 6. As shown in Figure 6a,b, fine graphite particles exist in the form of flake-type 2D sheets. The assembled graphite has relatively spherical shapes formed by the aggregation of fine graphite particles owing to the binder. The particle size increased to 17.5 μm based on the d50 criteria, compared with the size of fine graphite particles (Figure S1). Thus, it is validated that assembled graphite with a controlled shape and size was successfully manufactured.

The changes in tap density and BET-specific surface area before and after granulation are listed in Table 2. Compared with the fine graphite particles (NG1.2 and NG5), the assembled graphite (SG-series) exhibits a reduced specific surface area. The nm-scale pores detected in BET analysis accelerate the electrolyte decomposition reaction, reducing Coulombic efficiency and cycle stability [36,37]. Meanwhile, the tap density increased in all assembled graphite samples compared with fine graphite particles, with a maximum increase of 37% (SG60; 0.3142 g/mL). A high tap density implies a higher Li^+^ storage site in limited space, which is crucial for improving the energy density [38]. Therefore, assembled graphite can be applied as a promising anode material in terms of its high efficiency and high energy density.

### 3.3. Electrochemical Characteristics of Assembled Graphite

**Figure 7:**
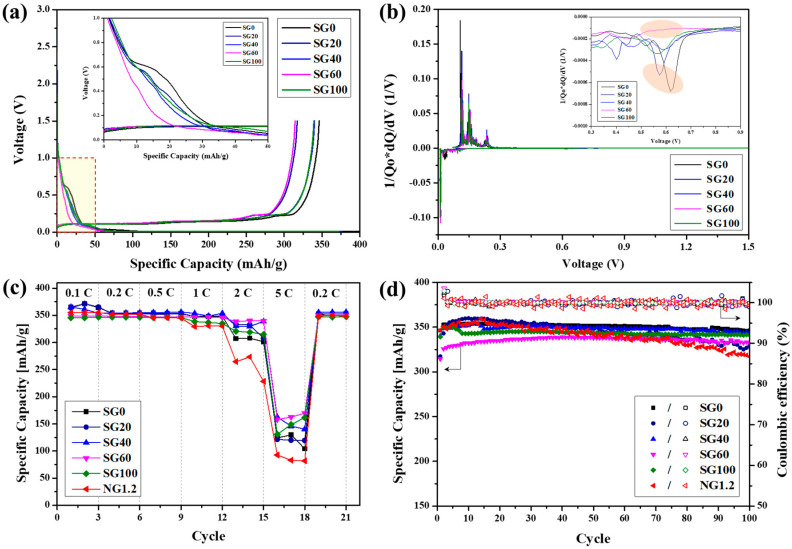
(**a**) First galvanostatic charge/discharge profiles of assembled graphite at 0.2 C (inset image; zoomed-in 0–1 V range); (**b**) differential capacity (dQ/dV vs. V) curves of assembled graphite at the 1st cycle (inset image; zoomed-in 0.3–0.9 V range); (**c**) rate performance test of assembled and fine graphite; (**d**) cycling performance of assembled and fine graphite (solid and open symbols indicate the specific capacity and Coulombic efficiency, respectively).

The galvanostatic charge and discharge profiles of the assembled graphite corresponding to the first cycle are shown in Figure 7a, and Table 3 summarizes the first cycle charge/discharge capacity and initial Coulombic efficiency (ICE). ICE increased as the open porosity calculated in Table 1 and Figure 3a decreased. SG60, which had the lowest open porosity at 0.94%, exhibited the highest ICE of 92.3%. The decrease in ICE originated from the irreversible capacity lost during the formation of the SEI layer [31,32,33]. Therefore, open pores accelerated the electrolyte decomposition reaction. Peaks related to SEI layer formation occurred in the 0.6–0.7 V range during the first charge profile, as depicted in Figure 7a [39,40]. A more detailed comparison of the peak intensity through differential capacity analysis (dQ/dV) is shown in Figure 7b. Based on the results, the most distinct peaks were observed in SG0 and SG20 (86.4% and 86.0%), which had the lowest ICEs. However, SG60, which had the highest ICE, only showed a slight peak, validating the effective suppression of the electrolyte decomposition reaction.

The rate performance test of the assembled and fine graphite particles was measured at scan rates of 0.1, 0.2, 0.5, 1, 2, and 5 C, respectively, as shown in Figure 7c. Following a pre-cycle (three cycles at 0.1 C), measurements were performed at each c-rate for every three cycles. NG1.2, the fine graphite particle with the smaller particle size, was selected for comparison to comprehensively examine the granulation effect. As presented in Figure 7c and Table S2, all the assembled graphite (SG-series) showed an improved rate capability compared with fine graphite particles. At 5 C/0.2 C, the capacity retention of NG1.2 was just 24.4%, the poorest among the samples. NG1.2 has limited sites for Li^+^ intercalation because the flakes aligned in a uniaxial direction during rolling [11,12,13]. However, the assembled graphite was less impacted by rolling effects because its flake pieces were assembled into a spherical shape without a preferred orientation, enabling multidirectional Li^+^ intercalation [20,21]. The superior rate capability of assembled graphite originated from this relatively advantageous structure for Li^+^ transport. Among the assembled graphite, SG60 exhibited the best rate capability, with a capacity retention of 47.0% at 5 C/0.2 C. This is because the binder pitch included in BSG60 had the highest carbon yield. Upon carbonization, the binder pitch transformed into amorphous carbon, which, owing to its low crystallinity, contributed less to capacity, but it is an excellent material for rate capability [41]. As explained in Section 3.1, BSG60 had the highest carbon yield owing to its unique packing structure (Table 1, Figure 4), indicating that it had the highest proportion of residual amorphous carbon. Therefore, the rate capability was enhanced owing to the high proportion of amorphous carbon in the assembled graphite. The prepared SG60 also exhibited excellent electrochemical properties compared to commercial graphite (Table S3).

The cycle performance test was conducted at 0.2 C for 100 cycles and is illustrated in Figure 7d. The assembled graphite showed excellent cycle stability, with over 100% capacity retention and 98% Coulombic efficiency after 100 cycles (Table 4). In contrast, NG1.2 exhibited a relatively low capacity retention of 91.5%. The continuous capacity fading of NG1.2 resulted from the formation of a thick and unstable SEI layer caused by its high specific surface area, as indicated in Table 2. This is evidenced in the EDS mapping image. Meanwhile, a slight initial increase in capacity was observed for the assembled graphite. This is common in materials with high porosity, resulting from gradual electrolyte infiltration [42,43].

**Figure 8:**
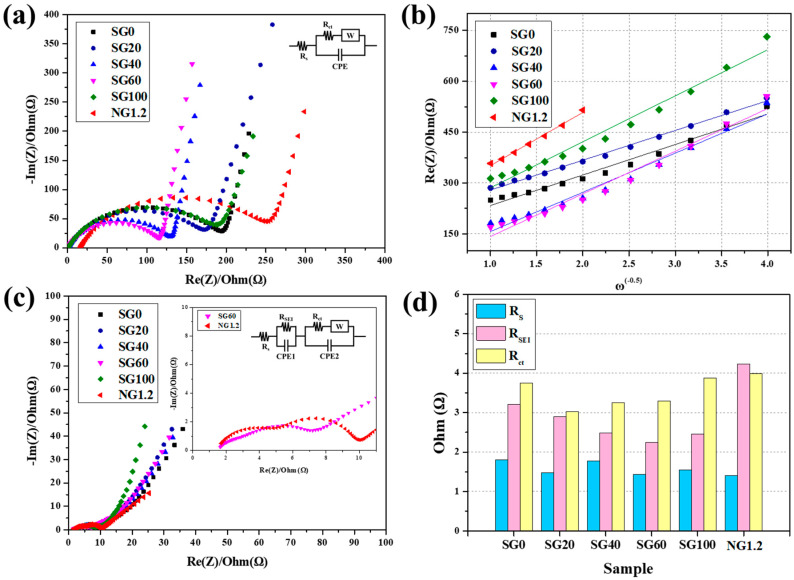
(**a**) Nyquist plots of the assembled and fine graphite before the cycle; (**b**) relationship between Re(Z) and the inverse square root of the angular speed (ω-0.5) in the low-frequency region; (**c**) nyquist plots of assembled and fine graphite after 10 cycles; (**d**) classified resistance of the electrode after 10 cycles.

To better understand the electrochemical behavior, we performed EIS measurements. Nyquist plots before and after 10 cycles are illustrated in Figure 8a and Figure 8c, respectively. The related EIS parameters are listed in Table 5. R_s_, R_SEI_, and R_ct_ represent the electrolyte resistance, resistance due to the formation of an SEI layer at the electrode interface, and charge-transfer resistance, respectively [44]. As shown in Figure 8a, the Nyquist plots of the pre-cycle samples comprised a semicircle and straight line in the high-frequency and low-frequency regions, respectively. The diameter of the semicircle is related to the charge-transfer resistance (R_ct_) at the electrode–electrolyte interface. Assembled graphite exhibited lower R_ct_ values compared with NG1.2, indicating improved accessibility for Li^+^ to the electrode. Among the assembled graphite, SG60 had the lowest R_ct_ value of 119.3 Ω, implying that it had the most favorable structure for Li^+^ transport. This is consistent with the rate performance test results shown in Figure 7c. The diameter of the first semicircle in the high-frequency region of the Nyquist plots after 10 cycles, shown in Figure 8c, represents R_SEI_, and the calculated values are presented in Figure 8d and Table 5. NG1.2 exhibited the highest RSEI value (4.23 Ω), which may be related to its poor cycling characteristics, as shown in Figure 7d.

The diffusion coefficient for *Li*^+^ (*D_Li_*^+^) was calculated to further investigate the electrochemical kinetics. The Warburg factor (σ) was calculated as follows [45,46]:

(4) $$Z^{\prime }=R_{1}+R_{ct}+{\sigma \omega }^{\left(-\frac{1}{2}\right)}$$

The value of σ was determined by utilizing the slope of linear fitting of the real part of the impedance (Re(Z)) versus the inverse square root of the angular frequency (ω-0.5), as shown in Figure 8b. The *D_Li_*^+^ was calculated based on the obtained σ using Equation (5) [45,46]:

(5) $$D_{{Li}^{+}}=\frac{R^{2}T^{2}}{{2A}^{2}n^{2}F^{4}C^{2}\sigma^{2}}$$

where *R* denotes the gas constant, *T* symbolizes the absolute temperature, *A* refers to the surface area of the electrode, *F* is Faraday’s constant, and *C* is the molar concentration of *Li*^+^ in the solution. The calculated *D_Li_*^+^ values are listed in Table 6. NG1.2 exhibited the lowest *D_Li_*^+^ value of 9.2007 × 10^−13^, indicating that Li^+^ diffusion within the electrode was more favorable in the assembled graphite.

**Figure 9:**
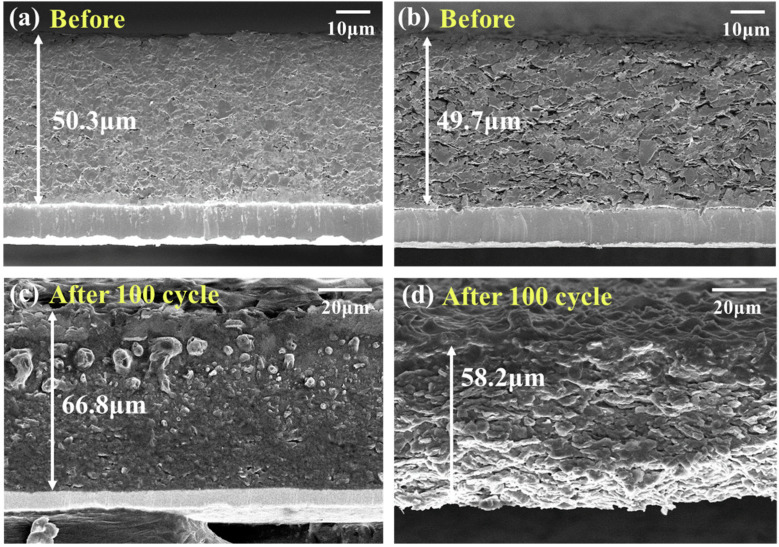
FE-SEM images of electrode cross-sections; (**a**) before cycling for NG1.2, (**b**) before cycling for SG60, (**c**) after 100 cycles for NG1.2, (**d**) after 100 cycles for SG60.

**Figure 10:**
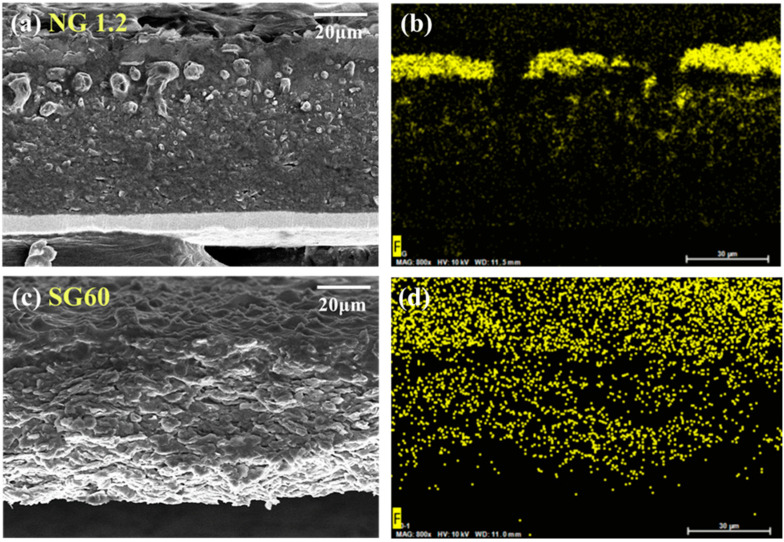
FE-SEM and EDS mapping images of electrode cross-sections after 100 cycles; (**a**,**b**) NG1.2, (**c**,**d**) SG60.

FE-SEM images of the electrode cross-sections before and after 100 cycles are displayed in Figure 9. In Figure 9a, NG1.2 shows densely packed particles in a flake-like form owing to their uniaxial directional compression during rolling. In contrast, as shown in Figure 9b, SG60 maintained micrometer-scale voids between particles, even after rolling, owing to its controlled morphology. Such voids facilitate electrolyte infiltration, which is correlated with the excellent rate capability of SG60. After 100 cycles, the electrode thicknesses for NG1.2 and SG60 were 66.8 µm and 58.2 µm, respectively, displaying expansion rates of 32.8% and 17.1%, respectively. When a lithium-ion battery (LIB) undergoes charge/discharge process, the intercalation/deintercalation of lithium ions into/from the graphite lattice causes repeated expansion and contraction of graphite. Stresses are inevitably generated in electrodes by such periodic volume changes and are probably accumulated during the cycling process. For this reason, volume expansion of the electrode occurs as shown in Figure 9, which is more critical to the NG1.2 electrode, which has more dense packing [47]. This difference in electrode expansion behavior originated from the structural features of the two materials. Based on numerous studies, the closed pores formed within the particles absorb the volume expansion owing to repeated charging and discharging, thereby enhancing cycle stability [34,35]. Therefore, the impact resulted from the closed pores that naturally occurred in the granulation process of SC60. EDS mapping was performed to further examine the distribution of the SEI layer after 100 cycles. The F mapping images, representing major elements of the SEI layer, are shown in Figure 10. In Figure 10a,b, NG1.2 shows a thick, localized accumulation of the SEI layer on the surface owing to its large specific surface area and dense particle packing. This resulted in a poor rate capability and low cycle stability. However, SG60 exhibited a uniform and stable formation of the SEI layer across the surface and the interior of the electrode.

## 4. Conclusions

In this study, assembled graphite electrodes were manufactured by utilizing fine graphite particles. Assembled graphite with differing pore characteristics was prepared by controlling the particle size distribution of the fine graphite particles. All assembled graphite samples exhibited reduced specific surface areas, increased tap density, and improved electrochemical properties compared with those of fine graphite particles. The most electrochemically superior assembled graphite was the SG60 electrode, which exhibited the lowest total porosity of 27.45% and the highest block carbon yield (90.22 wt%). The SG60 electrode showed the highest ICE of 92.3% owing to its low open porosity of 0.94% and also showed the lowest RSEI values after 10 cycles. Furthermore, the SG60 electrode exhibited only a 17.1% electrode expansion rate after 100 cycles at 0.2 C, further validating its vastly superior structural stability compared with fine graphite particles (with an electrode expansion rate of 32.8%). Finally, SG60 exhibited the highest rate capability owing to its multidirectional increase in Li^+^ intercalation sites and high amorphous carbon content. The granulation process proposed in this study could enhance properties by only controlling material composition, without involving complex procedures. Therefore, it is expected to be highly utilizable in the existing secondary battery anode material industry.

## Figures and Tables

**Table 1:** Density of the graphite block measured using various analysis methods, calculated porosity, and carbon yield of the graphite block after carbonization.

Sample Name	d_B_^(1)^ (g/mL)	d_A_^(2)^ (g/mL)	d_T_^(3)^ (g/mL)	Closed Porosity (%)	Open Porosity (%)	Total Porosity (%)	Yield (wt%)
BSG0	1.51	1.67	2.1931	21.41	9.56	30.96	87.89
BSG20	1.56	1.62	2.1871	25.16	3.37	28.54	89.63
BSG40	1.57	1.63	2.1900	24.84	3.55	28.39	89.99
BSG60	1.58	1.60	2.1848	26.51	0.94	27.45	90.22
BSG100	1.57	1.62	2.1936	25.50	2.90	28.40	89.98

^(1)^ d_B_: Bulk density, ^(2)^ d_A_: Apparent density, ^(3)^ d_T_: True density.

**Table 2:** Tap density and BET-specific surface area of fine and assembled graphite.

Sample Name	d_Tap_^(1)^ (g/mL)	S_BET_^(2)^ (m^2^/g)
NG1.2	0.2299	10.96
NG5	0.2656	12.13
SG0	0.3028	8.68
SG20	0.3053	8.59
SG40	0.2895	9.10
SG60	0.3142	8.51
SG100	0.2716	9.75

^(1)^ d_Tap_: tap density, ^(2)^ S_BET_: specific surface area.

**Table 3:** First cycle charge-discharge capacity and initial coulombic efficiency (ICE) of assembled and fine graphite electrodes.

	1st Charge Capacity (mAh/g)	1st Discharge Capacity (mAh/g)	I.C.E (%)
SG0	400.6	346.2	86.4
SG20	368.8	317.2	86.0
SG40	376.8	340.1	90.3
SG60	340.8	314.5	92.3
SG100	372.3	339.2	91.1
NG1.2	395.7	346.8	87.6

**Table 4:** Charge–discharge capacity and capacity retention after 100 cycles for assembled and fine graphite.

	1st Discharge Capacity (mAh/g)	100th Discharge Capacity (mAh/g)	Capacity Retention after 100 Cycles (%)
SG0	346.2	346.1	100.0
SG20	317.2	329.6	103.9
SG40	340.1	344.4	101.3
SG60	314.5	332.3	105.7
SG100	339.2	340.9	100.5
NG1.2	346.8	317.2	91.5

**Table 5:** EIS parameters of assembled and fine graphite before and after 10 cycles.

Sample	Before the Cycle		After 10 Cycles		
	R_S_ (Ω)	R_ct_ (Ω)	R_S_ (Ω)	R_SEI_ (Ω)	R_ct_ (Ω)
SG0	1.42	200.6	1.80	3.21	3.75
SG20	1.32	179.4	1.47	2.89	3.02
SG40	1.31	133.5	1.78	2.48	3.25
SG60	1.13	119.3	1.43	2.24	3.30
SG100	1.08	199.0	1.55	2.46	3.88
NG1.2	15.9	249.3	1.40	4.23	3.99

**Table 6:** Li-ion diffusion coefficient of assembled and fine graphite.

Sample	SG0	SG20	SG40	SG60	SG100	NG
*Di_Li_*^+^ (cm^2^ s^−1^)	2.8216 × 10^−12^	1.8314 × 10^−12^	2.0192 × 10^−12^	1.7675 × 10^−12^	1.1183 × 10^−12^	9.2007 × 10^−13^

## Data Availability

Data are contained within the article.

## Supplementary Materials

The following supporting information can be downloaded at: https://www.mdpi.com/article/10.3390/ma16216896/s1, Figure S1: Particle size distribution of fine and assembled graphite; Table S1: Tap density based on the fraction of small graphite particles; Table S2: Average capacity and capacity retention based on the C-rate; Table S3: Electrochemical properties of assembled graphite, NG1.2 and commercial graphite: discharge capacity, first cycle Coulombic efficiency, and Retention rate. Reference [48] are cited in the supplementary materials.
