# Supplementary material for: Optimization of Pore Characteristics of Graphite-Based Anode for Li-Ion Batteries by Control of the Particle Size Distribution
Source: Materials (Basel). 2023 Oct 27;16(21):6896. doi: 10.3390/ma16216896 (PMC10650451; doi:10.3390/ma16216896)
Supplement: Supplementary file 1 [file materials-16-06896-s001.zip › materials-2640654-supplementary.pdf]

Supplementary Information

Table S1. Tap density based on the fraction of small graphite particles

Xs	0	10	20	30	40	50	60	80	90	100
Tap density (g/ml)	0.27	0.28	0.28	0.32	0.35	0.32	0.31	0.31	0.26	0.23

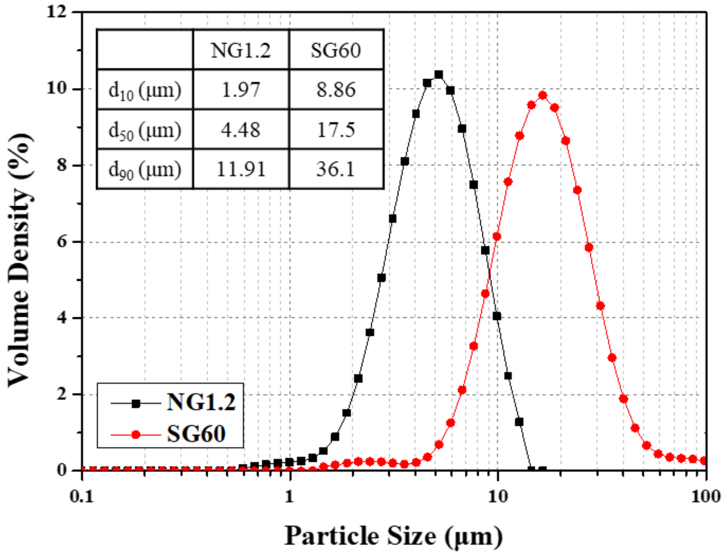

Figure S1. Particle size distribution of fine and assembled graphite.

Table S2. Average capacity and capacity retention based on the C-rate

	0.1C (mAh/g)	0.2C (mAh/g)	0.5C (mAh/g)	1C (mAh/g)	2C (mAh/g)	5C (mAh/g)	0.2C* (mAh/g)	5C/0.2 C (%)	0.2C*/0.2 C (%)
SG0	366.8	354.0	353.5	347.8	305.4	119.3	352.4	33.7	99.55
SG20	366.8	352.9	352.3	348.5	324.4	120.2	352.9	34.1	100.0
SG40	360.0	354.4	355.5	352.0	332.8	149.6	355.8	42.2	100.4
SG60	348.8	347.9	347.2	346.2	339.3	163.4	350.1	47.0	100.6
SG100	346.0	346.4	345.9	336.8	318.1	147.0	347.1	42.5	100.2
NG1.2	355.1	352.0	345.3	329.9	255.4	85.8	348.9	24.4	99.13

Table S3. Electrochemical properties of assembled graphite, NG1.2 and commercial graphite: discharge capacity, first cycle Coulombic efficiency, and Retention rate.

	Capacity at the first cycle ($mAhg^{-1}$)			Retention Rate
	Discharge	Irreversible	C.E. (%)	5C/0.1C (%)
SG60	314	26	92.3	46.8
NG1.2	346	49	87.6	24.2
Commercial Graphite[1)]	344	46	88.2	30.0

[1)]Han, Y.-J.; Kim, J.; Yeo, J.-S.; An, J.C.; Hong, I.-P.; Nakabayashi, K.; Miyawaki, J.; Jung, J.-D.; Yoon, S.-H. Coating of graphite anode with coal tar pitch as an effective precursor for enhancing the rate performance in Li-ion batteries: Effects of composition and softening points of coal tar pitch. Carbon 2015, 94, 432-438.
